# Circulating MicroRNAs as Biomarkers in Biliary Tract Cancers

**DOI:** 10.3390/ijms17050791

**Published:** 2016-05-23

**Authors:** Pablo Letelier, Ismael Riquelme, Alfonso H. Hernández, Neftalí Guzmán, Jorge G. Farías, Juan Carlos Roa

**Affiliations:** 1 School of Health Sciences, Universidad Católica de Temuco, Manuel Montt 56, 4813302 Temuco, Chile; pletelier@uct.cl (P.L.); alfonso.hernandez@uct.cl (A.H.H.); nguzman@uct.cl (N.G.); 2 Molecular Pathology Laboratory, Department of Pathological Anatomy, School of Medicine, Universidad de La Frontera, Avenida Alemania 0458, 3rd Floor, 4810296 Temuco, Chile; ismael.riquelme.contreras@gmail.com; 3 Scientific and Technological Bioresource Nucleus (BIOREN), Universidad de La Frontera, Avenida Francisco Salazar 01145, Casilla, 54-D Temuco, Chile; 4 Department of Chemical Engineering, Faculty of Engineering and Sciences, Universidad de La Frontera, 54-D Temuco, Chile; jorge.farias@ufrontera.cl; 5 Department of Pathology, Centre for Investigational Oncology (CITO), Advanced Centre for Chronic Diseases (ACCDiS), Pontificia Universidad Católica de Chile, Marcoleta 377, 7rd Floor, 8330024 Santiago, Chile

**Keywords:** biliary tract cancers, microRNAs, biomarkers

## Abstract

Biliary tract cancers (BTCs) are a group of highly aggressive malignant tumors with a poor prognosis. The current diagnosis is based mainly on imaging and intraoperative exploration due to brush cytology havinga low sensitivity and the standard markers, such as carcinoembryonic antigen (CEA) and carbohydrate 19-9 (CA19-9), not having enough sensitivity nor specificity to be used in a differential diagnosis and early stage detection. Thus, better non-invasive methods that can distinguish between normal and pathological tissue are needed. MicroRNAs (miRNAs) are small, single-stranded non-coding RNA molecules of ~20–22 nucleotides that regulate relevant physiological mechanisms and can also be involved in carcinogenesis. Recent studies have demonstrated that miRNAs are detectable in multiple body fluids, showing great stability, either free or trapped in circulating microvesicles, such as exosomes. miRNAs are ideal biomarkers that may be used in screening and prognosis in biliary tract cancers, aiding also in the clinical decisions at different stages of cancer treatment. This review highlights the progress in the analysis of circulating miRNAs in serum, plasma and bile as potential diagnostic and prognostic markers of BTCs.

## 1. Introduction

Biliary tract cancers (BTCs) are a group of highly aggressive malignant tumors with a poor prognosis; therefore, most patients are diagnosed at an advanced stage when the tumor is unresectable or metastatic [1,2]. In recent years, new strategies have been sought for the early diagnosis of BTCs, with the most studied being biochemical markers that have proven most relevant in complementing diagnosis and prognosis: total bilirubin, alanine aminotransferase and tumor markers [3,4]. Serum levels of carcinoembryonic antigen (CEA) and carbohydrate 19-9 (CA19-9) are usually elevated and serve as a standard for clinical diagnosis, although none has sufficient sensitivity or specificity to be used in a differential diagnosis and early stage detection [5,6]. In view of these limitations, diagnosis occurs by pathological examination of the biopsy obtained either via endoscopic retrograde cholangiopancreatography (ERCP), or percutaneous transhepatic cholangiography (PTC), or ultimately, during surgical exploration [7]. Thus, better non-invasive methods that can distinguish between normal and pathological tissue are needed. MicroRNAs (miRNAs) are small non-coding RNA molecules of ~20–22 nucleotides, which play an important regulatory role in the mRNA silencing of genes [8] involved in carcinogenic processes, such as development [9], cell cycle and apoptosis [10,11,12], directly affecting tumor progression [13]. For this reason, miRNAs are promising therapeutic targets and diagnostic biomarkers [14]. A biomarker is a biological indicator that should be stable in fluids, accessible, disease-specific [15] and easy-to-measure in the body in order to predict the incidence of outcome or disease [16]. Many studies have shown that miRNAs are a novel class of biomarkers to diagnose human cancers [17,18]. The analysis and determination of circulating miRNAs in multiples biological fluids, such as serum, urine, saliva and bile, could be an alternative to the determination of proteins [19,20]. Recently, accumulated evidence has shown a detectable and deregulated expression of tissues and circulating miRNAs in BTCs, closely associated with prognosis and diagnosis in these patients [21,22,23,24]. This review focuses on a general description of bile tract cancers, the biogenesis of miRNAs and their regulatory mechanisms and the progress of circulating miRNAs as diagnosis and prognosis biomarkers for BTCs.

## 2. Brief Description of Biliary Tract Cancers

Biliary tract cancers include intra- and extra-hepatic cholangiocarcinoma (CCA), ampullary carcinoma (AC) and gallbladder cancer (GBC) [25,26]. Many risk factors are strongly associated with BTCs, such as gallstones, chronic inflammation, parasitic infections, biliary duct cysts, hepatolithiasis, primary sclerosing cholangitis (PSC), chronic liver disease, gallbladder polyps, in addition to non-modifiable risk factors, such as ethnic background, increasing age, female gender, congenital biliary abnormalities and genetic factors. Other lifestyle factors, like obesity, multiparity, cigarette smoking, alcohol consumption and hepatitis B or hepatitis C infection, are also strongly related to this pathology [27,28,29]. Most BTC patients present symptoms similar to those of patients with benign biliary diseases (BBDs) [30]. These neoplasms exhibit dismal long-term survival outcomes, and although these malignancies are anatomically related, each has a distinct molecular biology and clinical and epidemiological presentation [31]. GBC is the most common malignancy of the biliary tract and the fifth most common malignant tumor of the digestive tract. It was first described in 1777 by German surgeon Maximillian Stoll [32,33]. The evolution of this disease is usually asymptomatic, resulting in a late diagnosis with poor survival [32]. The epidemiology of the disease is highly heterogeneous; populations at low risk for GBC are in northern Europe and among the non-Hispanic white population of the United States. The high-risk populations are found in the Andean countries, particularly Chile, Bolivia and among Hispanic and Indian populations in North America with incidence rates of 3.5–7.0 and 10–15 cases per 100,000 habitants in men and women, respectively [34]. CCA is a malignant tumor arising in the bile duct epithelium, usually diagnosed late, when surgery is no longer an option [35,36]. Anatomically, CCA is divided into intrahepatic cholangiocarcinoma (iCCA) and extrahepatic cholangiocarcinoma (eCCA), which is divided intoperihilar (pCCA) and distal (dCCA) tumors [37,38,39]. It was initially described by Durand-Fardel in 1840 [37]. Its frequency is less than 3% of gastrointestinal malignancies, and its incidence in the U.S. is 1.0/100,000 per year [37]; however, it has a significant impact on public health in Southeast Asian countries (incidence of 96 per 100,000) [40]. AC is a relatively uncommon tumor, representing 0.2%–0.5% of gastrointestinal malignancies; the overall incidence rate is 0.49 per 100,000 individuals in the U.S. [41]. Most ampullary carcinomas are adenocarcinomas [42], and three epithelial types can be found in this area, originating in either the duodenal mucosa, the bile duct or the pancreatic duct [43]. Prognosis depends on histological typing of the tumor and its clinical stage. Studies report a good prognosis if the tumor is limited to the duodenal mucosa without invasion into adjacent organs [43]. The mortality rate of BTCs has increased considerably because of frequent metastasis, high recurrence rates and poor response to chemotherapy and radiation therapy when surgical resection is possible. Long-term survival depends heavily on lymph node involvement, negative surgical resection margins and tumor differentiation [44,45]. The method for initial study in BTC is abdominal ultrasonography ; however, the sensitivity of this technique to detect early lesions is limited [46]; therefore, most patients are diagnosed at an advanced stage when the tumor is unresectable or metastatic [1,2]. Other high technology, such as computed tomography (CT), cholangioscopy, endoscopic ultrasound (EUS) and magnetic resonance imaging (MRI), are expensive and usually of limited use in the general population, and it is uncommon in the diagnosis of small (early-stage) lesions [47,48,49].

## 3. MicroRNAs Biogenesis and Their Regulatory Mechanisms

The accumulated evidence indicates that the miRNAs are involved in the regulation of cell differentiation, proliferation and apoptosis [50,51], as well as in the regulation of genes associated with cancer formation [52]. miRNAs may function as tumor suppressors or oncogenes [52,53,54]. When miRNAs are overexpressed in cancers, they usually function as oncogenes by negatively regulating tumor suppressor genes (pro-apoptotic or anti-proliferative roles). Conversely, when miRNAs are repressed in cancers, they function as tumor suppressor genes and may inhibit cancer cells by regulating oncogenes [55,56,57,58].

During biogenesis, miRNAs are initially transcribed by RNA polymerase II or III, as a primary transcripts at the nucleus (pri-miRNA) that contains a stem-loop sequences of ~80-nts [59,60,61]. miRNA genes are located in intergenic regions, intronic regions of known genes, transcribed individually or in tandem with polycistronic sequences [62,63], allowing multiple miRNAs to be expressed simultaneously. This co-expression could ensure negative feedback within the same pathway [64]. A lower number of miRNAs are expressed along with their own promoter regions [65] and regulated by many transcription factors [66]. The pri-miRNA is converted into a ~60-nt precursor or pre-miRNA by nuclease Drosha/DGCR8 [67]. Then, the pre-miRNA is exported from the nucleus into the cytoplasm by exportin 5 (XPO5) and Ran-GTP [68]. This pre-miRNA is then processed to the miRNA/miRNA* duplex in the cytoplasm by its interaction with Dicer ribonuclease and the cofactor TRBP, which finally form the mature miRNA [69]. Finally, one strand is incorporated into the effector RNA induced silencing complex (RISC), which couples this mature sequence to target mRNAs. On the other hand, the passenger strand (miRNA* strand) is degraded by the RISC complex [70,71,72]. However, in some cases, miRNA* strands are retained, having a regulatory role as a mature miRNA [73]. There are alternative biogenesis pathways that differ in processing steps and the enzymes responsible. Particularly, many miRNAs can be processed by Ago-2 in a Dicer-independent manner [74]. In addition, mirtrons are a type of microRNAs that is located in the intron regions and is digested via the spliceosome [73,75] and/or in a splicing-independent manner, these being so-called “simtrons” [76]. The multiple biogenesis processes could be related to different development stages in cells [76]. Following transcription, miRNAs can be modified at the post-transcriptional level, potentially affecting miRNA stability and the efficiency of miRNA and miRNA* [66].

miRNAs induce gene silencing by binding to target mRNAs in complementary sequences (miRNA recognition element) located in the 3′ untranslated region (UTR) of the mRNA, using a short sequence of approximately 2–8 nucleotides (the seed region) at the 5′ end of the miRNA, allowing miRNA targeting to have more than one mRNA. This interaction inhibits the translation process and protein synthesis, resulting in a complex regulatory network [12,52,77]. In addition, some reports indicate that miRNAs can also be joined to the 5′ UTR and the coding region of the mRNAs [78,79]. However, in these regions, the effectiveness in repressing the translation decreases, probably due to the reduced stability in the miRNA-RISC/mRNA complex produced when the ribosomes interact [80]. Furthermore, complex stability depends on the sequence of the binding site, the number of target sites within mRNA, the local structure of RNA and the distance between target sites [81,82,83].

The mature miRNA attached to the RISC complex is the complex effector of gene silencing, which is presented to enable binding by pairing bases to target sites in the RNA. If there is high complementarity between miRNA/mRNA, the mRNA is cleaved by endonuclease Ago2, possibly inclusion bodies and RNA processing (P-bodies) [77,84] and, if imperfectly complementary, only silencing mRNA translation occurs [12,85].

Scientific evidence has shown that miRNAs expression can be controlled by various molecular events, including alterations in genome location, epigenetic changes, transcriptional deregulations and alterations in miRNA biogenesis. Several microRNA genes are located near breakpoint regions, which can present loss of heterozygosity (LOH), amplifications, deletions or mutations [86,87,88]. Moreover, DNA methylation is a potent regulator of miRNA expression and histone modifications and is a frequent event in cancer [89,90,91]. Aberrant DNA methylation in the promoter regions produced the silencing of miRNAs, modifying the expression of tumor suppressor miRNAs [92,93,94] and oncogenic miRNAs [95,96]. Moreover Dicer, Drosha and XPO5 inactivation have been shown to induce an altered biogenesis and, therefore, a significant reduction of miRNA levels, leading to an aberrant expression in several cancers [97,98,99,100,101]. Some proteins (HnRNPA1, SMAD1 and SMAD5) have been involved in the regulation of miRNAs precursors, modifying subsequently their expression [102]. A defect in XPO5 also affects the transportation of pre-miRNAs, provoking the nuclear retention of these precursors [66]. Additional evidence has demonstrated that some dietary compounds, such as folate, retinoids and curcumin, modify the microRNAs expression levels, acting as protective factors [103]. Other factors, such as the hormonal status [104] and hypoxic conditions, can also modify miRNA expression [105].

## 4. MicroRNAs in Extracellular Vesicles

**Figure 1:**
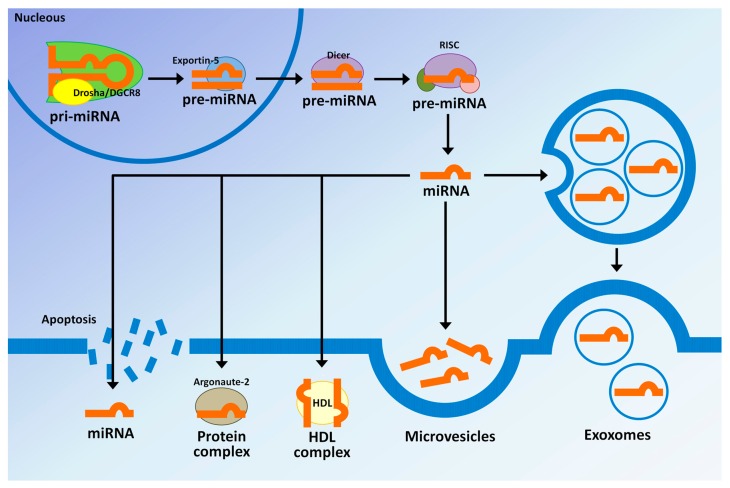
Origin mechanism of circulating microRNAs. MicroRNAs are exported from cells into circulation by free vesicles (active secretion via exosomes and microvesicles) and are associated with ribonucleoproteins, particularly with Argonaute (AGO) proteins. miRNAs have also been found in apoptotic bodies and associated with complexes with high density lipoprotein (HDL).

One of the most remarkable aspects of miRNAs is their high stability outside the intracellular environment; this aspect is of interest, as potential biomarkers of clinical use (reviewed by Moldovan *et al*. [106]). The mechanisms by which the circulating miRNAs reach the circulation are not fully understood; however, one of the mechanisms through which they originate is active secretion via extracellular vesicles (EVs) [107]. Extracellular RNAs (exRNA) may exist in essentially four forms: freely circulating, bound to specific proteins, associated with lipoproteins or enclosed in extracellular vesicles (EVs) (Figure 1) [108]. These include exosomes and microvesicles, which are formed by a lipid membrane and contain specific molecules, constituting a communication system between different cell types in physiological and pathological processes [109,110]. These EVs have been found in several cell types, including macrophages, endothelial cells, monocytes, leukocytes, tumor cells [111] and even in embryonic stem cells [111]. Exosomes are distributed in various body fluids, including human bile, causing them to come into contact with neighboring or distant cells and then deliver the miRNA content [110,112], exhibiting paracrine effects on tumor growth [113,114].

The molecular structure of an EV is very similar to the parental cell or tissue and may contain other molecules, such as receptors, adhesion molecules, proteins, lipids, growth factors, proteases and exRNAs [115,116]. The diversity of exRNA species in EVs is extensive, and according to the different analyses, it is known that miRNA and piwiRNA are the most abundant RNA species, with a lower percentage having been found of pseudo-genes, LncRNAs, tRNAs and mRNAs [117]. miRNAs are secreted both in EVs and in a non-vesicular form. The loading of miRNAs into EVs is controlled by heterogeneous nuclear ribonucleoprotein (hnRNP) A2B1, which recognizes the EXOmotif (GGAG tetranucleotide) of miRNAs (miRNAs into exosomes attached to specific motifs) and regulates the transfer into EVs. Heterogeneous nuclear ribonucleoprotein A2/B1(hnRNPA2B1) inside the EVs is SUMOylated; this post-translational modification is necessary for the loading of miRNAs into EVs [118]. miRNA disposal into the EV can be facilitated by the addition of non-templated nucleotides to the 3′end of the miRNA [119]. Current evidence suggests that the miRNAs enclosed in EVs can increase their half-life in circulation, averting the degradation from RNases present in blood [120]. The EV-mediated transfer of miRNAs has been shown to have several physiological roles, for example in the immune response [121]. The selective removal of miRNAs in EVs is also a regulation mechanism, especially the removal of tumor suppressor miRNA in cancer [122,123]. Although it was believed that vesicle encapsulation was the principal mode of stabilization for ex-miRNAs [124], increasing evidence shows that the vast majority of small RNAs exported by mammalian cells are principally associated with ribonucleoproteins more than vesicles [125], particularly with Argonaute (AGO) proteins [126,127]. miRNAs have also been found in apoptotic bodies [128] and associated in complexes with high-density lipoprotein (HDL) [129,130]. It has been demonstrated that these lipoproteins transport endogenous miRNAs and deliver them to the recipient cells; this important discovery suggests that some of the biological effects of HDL could be mediated by these miRNAs. The functional uptake of miRNAs has only been observed in membrane associated and lipoprotein transporters, but not for miRNA/protein complexes. The functional uptake is usually defined as the capability of the exogenous miRNA, transferred within the exosome, to selectively influence the gene expression in the host cell [129].

## 5. miRNAs as Biomarkers in Biliary Tract Cancer: miRNAs in Blood (Plasma and Serum), Bile and Gallstones

### 5.1. Plasma/Serum miRNA Panels as Biomarkers

Meng *et al.* described the first approach by which miRNAs could be used as biomarkers in cholangiocarcinoma. They found that miR-21 and miR-200b are related to sensitivity/resistance to gemcitabine [131]. Various current publications have reported on the potential clinical application of circulating miRNAs in BTCs for diagnosis and prognosis; most have focused on CCA, with little information being provided on GBC and ampullary adenocarcinoma. Li *et al.* [132] analyzed the expression of circulating miRNAs in patients with GBC, finding that miR-21, miR-187 and miR-202 were upregulated; by contrast, let-7a, miR-143 and miR-335 were downregulated between GBC patients and healthy controls (*p* < 0.05), showing a consistent expression in tissue and blood samples. Moreover, a significant relationship was found between the differential expression of three miRNAs (miR-187, miR-143 and miR-202) and lymphatic metastasis/TNM [132]. These data are consistent with the prior report performed by Kishimoto *et al.* [30], where expression levels of miR-21 in patients with BTCs (including intrahepatic cholangiocarcinoma, bile duct cancer, gallbladder cancer and cancer of the ampulla of Vater) were significantly higher than in healthy controls or in patients with BBDs, with the area under the curve (AUC) for plasma being 0.93 and 0.83, respectively (Table 1). The sensitivity and specificity was 85.1% and 100%, respectively, for healthy controls and 72.3% and 91.3% for BBDs [30]. In addition, the high expression of miR-21 was associated with the progression of TNM staging, and the diagnostic power increased by the combined use of CA19-9 and miR-21 for distinguishing between BTCs and healthy controls. Interestingly, circulating miR-21 expression decreased after surgery [30]. Furthermore, miR-21 is uniformly overexpressed in human CCA tissue and cell lines, possessing a sensitivity and specificity of 95% and 100% (AUC of 0.995), respectively, enough to differentiate CCA from normal tissues [22,133].

Plieskatt *et al.* [40] performed a comprehensive analysis of profiled miRNA expression levels in iCCA tumor tissue and matched plasma samples. The analysis of miRNAs in plasma was based on the aberrant expression observed in tissues. It was found that the expression of eight miRNAs (miR-483-5p, miR-505-3p, miR-874, miR-885-5p, miR-320b, miR-92b-3p, miR-1275 and miR-1307-3p) was detected exclusively in all iCCA plasma samples and not in controls plasma [40]. In addition, the aberrant microRNA expression in tissues was not similar to that observed in plasma, where fifteen of the highly deregulated miRNAs were only detected in tissues samples. This expression profile in plasma could be useful as a biomarker for the early detection of iCCA. Similarly, Wang *et al.* [134] studied the expression of miRNAs in tissue and matched plasma samples, reporting that miR-150 presented the most significant expression level and was upregulated in ICC plasma patients, with the opposite result being observed in tissue, where it was downregulated. No significant differences were found between the levels of miR-150 and age, gender or clinical stage of patients. Based on the ROC analysis, plasma miR-150 could be used to distinguish patients with iCCA from healthy volunteers (HVs), with the AUC being 0.764 (*p* < 0.010), sensitivity 80.6% and specificity 58.1%. In addition, the diagnostic value is increased when combined with the carbohydrate antigen 19-9 marker, improving patient screening [134]. miR-150, it has been reported, may function as oncogenes or tumor suppressors, is significantly upregulated (acts as an oncogene) in several types of cancers, e.g., in breast cancer [140] and gastric cancer [141], and is significantly downregulated (acts as a tumor suppressor) in colorectal cancer [142] and osteosarcoma [143]. There are no other studies correlating this microRNA with BTCs. Meanwhile, Silakit *et al.* [135] demonstrated that miR-192 levels were significantly higher in the pooled serum of CCA patients than in pooled HVs, with a sensitivity and specificity of 74% and 72%, respectively (AUC of 0.803). Moreover, miR-192 expression was significantly linked to lymph node metastasis and associated with a poor prognosis in CCA patients [135]. In addition, miR-200b expression was also significantly higher in CCA patients than HVs [135], but this miR-200b expression was relatively lower compared to miR-192. In tissues, a high expression of miR-192 was observed in 53% of tumor cases compared toadjacent non-tumor tissues; however, no significant difference was found [135]. Huang *et al.* [144] found that miR-224 was significantly upregulated in serum, as well as in cancer tissue from CCA patients compared to healthy controls. Furthermore, the human CCA cell lines transfected with a miR-224 mimic showed enhanced cell growth, invasiveness and migratory ability [144]. Kojima *et al.* [138] studied a large number of cases of gastrointestinal cancers, including BTCs (intrahepatic and extrahepatic bile duct, gallbladder, hilar bile duct and ampulla of Vater) and healthy controls. They found that 66 miRNAs showed statistical significance in the validation cohort (test cohort), among which 30 miRNAs were upregulated and 36 miRNAs were downregulated in BTCs. Expression of miR-125a-3p and miR-6893-5p showed the lowest *p*-values in a comparison of BTCs and HVs. In addition, miR-125a-3p was able to detect 32 of 33 patients (97.0%) in the test cohort [138]. Unfortunately, miR-125a-3p lacks some specificity because it was described as a reliable marker for pancreatic cancer, and a strong overlap is observed in the expression of this miRNA between these two types of cancers. At the same time, Que *et al.* [145] evaluated the expression partially of circulating miRNAs in AC. Four miRNAs (miR-17-5p, miR-21, miR-155 and miR-196a) related to pancreatic adenocarcinoma (PA) were selected to evaluate serum exosomal miRNA levels. No significant differences were found in the expression levels of miR-21 and miR-17-5p between PC patients and AC patients, although they tended to be lower in AC patients. Furthermore, expression of these miRNAs in AC tended to be higher than in healthy controls [145]. According to these results, none of the selected miRNAs reached statistical significance to discriminate between pancreatic cancer and BTCs.

Another study performed by Cheng *et al.* [139] found that circulating miR-106a is downregulated in CCA patients compared to BBD patients or healthy controls. Moreover, the serum level of miR-21 was higher in CCA patients than in BBD controls; however, no statistically-significant differences were found. The AUC of serum miR-106a for differentiating CCA patients from healthy controls was 0.89; the sensitivity and specificity for this marker were 81.6% and 85.0%, respectively. This result is moderate and higher than serum CA19-9. Meanwhile, downregulation of miR-106a in serum samples was associated with lymph node metastasis and poor prognosis in CCA patients [139].

### 5.2. Bile miRNA Panels as Biomarkers

Bile is a fluid produced by the liver and secreted into the intestine through biliary ducts to help digest fats. It is mainly composed of water, salts from biliary acids, compounds of glycolic and taurocholic acid and bile pigments (bilirubin), as well as proteins, electrolytes, mucus and various metabolites [146], such as miRNAs, which have been proposed as excellent biomarkers in the diagnosis of gastrointestinal tumors [147,148]. It has also been reported that extracellular vesicles are present in human bile and contain a large number of miRNAs [24], which makes these molecules very stable [114]. Li *et al.* [24] defined a novel biliary microvesicle-“laden” miR-based panel for CCA diagnosis, combining five miR markers (miR-191, miR-486-3p, miR-1274b, miR-16 and miR-484) that were able to differentiate between CCA from primary sclerosing cholangitis or other bile duct obstruction, with a sensitivity of 67% and a specificity of 96%. Considering the individual expression of miRNAs, miR-486-3p has a greater diagnostic value for CCA [24]. Similarly, Shigehara *et al.* [114] demonstrated that 10 of the 667 bile miRNAs studied (miR-9, miR-145*, miR-105, miR-147b, let-7f-2*, let-7i*, miR-302c*, miR-199a-3p, miR-222* and miR-942) were significantly more highly expressed in the malignant group composed of CCA and GBC patients than in the control group (patients with choledocholithiasis). Moreover, ROC analysis showed that the combination of two miRNAs (miR-9 and miR-145*) is a suitable diagnostic biomarker for BTCs [114].

A study conducted by Voigtlander *et al.* [149] revealed that patients with primary sclerosing cholangitis (PSC), a pathology that causes inflammation and obliterative fibrosis of the bile ducts of the liver, increased the risk of developing CCA, having distinct miRNA profiles in serum and bile. A serum validation analysis found significantly higher levels of miR-1281, miR-126, miR-26a, miR-30b and miR-122 in patients with PSC compared to patients with CCA. All of the validated circulating miRNAs were significantly higher in the abnormal cases than in HVs. By contrast, miR-412, miR-640, miR-1537 and miR-3189 expression was different between patients with PSC and PSC/CCA in bile samples [136], of which only miR-412 was upregulated [149]. Similarly, Bernuzzi *et al.* [137] also attempted to identify a miRNA panel for the diagnosis of PSC-related to CCA, but only in serum samples. It was found that miR-200c is significantly downregulated in PSC *versus* HVs, whereas miR-483-5p and miR-194 were upregulated in CCA compared to HVs. If we observe what occurs between CCA with PSC, as in the previous study, miR-222 and miR-483-5p were found to be upregulated in CCA *versus* PSC [137]. Patients with PSC and/or CCA have distinct miRNA profiles in serum and bile; thus, these miRNA expressions could possibly be used to detect an early lesion. However, in this study, Voigtlander *et al.* [149] could not use matched samples (blood and bile) from the same patients to establish this differentiation.

### 5.3. miRNA Biomarkers in Gallstones for the Identification of High-Risk Patients with Complications in the Biliary Tract

Gallstones are an important risk factor associated with GBC, being present in most (~85%) patients [150]. The expression profile of miRNAs in gallstones could represent a new way to assess the degree of injury that the epithelium could potentially present in these patients. The formation of gallstones occurs as a consequence of the excess of cholesterol precipitation in bile caused by a variety of factors that involve cholesterol absorption, biosynthesis and conversion [151]. Interestingly, Yang *et al.* [152] evaluated the miRNA expression in gallstone tissue, finding that miR-210, miR-200c, miR-194 and miR-192 expression was significantly upregulated and miR-133a and miR-891a expression significantly downregulated in patients with gallstones compared to those without gallstones [152]. The miRNA-200 cluster (miR-200a, miR-200b and miR-200c) has been associated with human GBC via epigenetic regulation [153] and contributes to bile duct proliferation in the cholestatic liver [154]. Additionally, a circulating miR-200b/429 cluster was shown to be significantly increased in the sera of biliary atresia suggesting its use as potential biomarkers [155], and the expression of miR-133 was consistently found decreased in tumors and GBC cell lines compared to normal gallbladder tissues [21]. However, in the study by Yang *et al.* [152], all patients included were confirmed as having no inflammation in the tissue.

Related studies on the synthesis of cholesterol and human gallstones have shown that the FXR/SHP pathway regulates the miR-34a expression and its target SIRT1, which is associated with gallstones because it participates in lipid metabolism and hepatic bile acid synthesis [156]. The farnesoid X receptor (FXR) is an important regulator of bile formation, controlling the secretion of phospholipids, bile salts, and is also associated with the regulation of liver X receptors (LXR), secretion modulators [157]. Recently, it was observed that the activation of FXR regulates SREBP-2 and miR-33 expressionand the regulation of their specific target genes [158]. miR-33 inhibits expression of the mRNAs ABCB11 and ATP8B1, which results in changes in bile secretion and bile recovery from the gallbladder [159]. In addition, ATP-binding cassette transporter A1 (ABCA1) is a major determinant of HDL-cholesterol levels, and its expression is regulated by miR-144 [160]; and miRNAs 122a and 422a may inhibit the human cholesterol 7a-hydroxylase mRNA (CYP7A1), which is intricately involved in regulating bile acid synthesis in the liver [161]. Several miRNAs have a major influence on cholesterol homeostasis, including miR-122 [162], miR-370 [163], miR-378/378* [164], miR-143, miR-27 [165], miR-335 [166], and miR-33a/b [167,168], miR-27a [169], miR-223 [170], miR-181a [171], miR-185 [172] and miR-223 [173]. The physicochemical imbalance between the major bile lipids produces hypersecretion of cholesterol in the bile, necessary to saturate the gallbladder and therefore determining individual predisposition to develop gallstones [174,175]. Thus, miRNAs in gallstones and/or deregulation of miRNAs involved in cholesterol homeostasis may be critical in the formation of gallstones, providing a new option for primary detection in subjects at risk ofdeveloping a malignancy or GBC development.

## 6. Discussion

The diagnosis of BTCs is mainly based on imaging, laparoscopic cholecystectomies and intraoperative exploration performed for cholelithiasis [150]. The examination of cells from the mucosa of the biliary tract can be performed through a brush cytology, which has a high specificity, but a low sensitivity, mainly due to the anatomical location of the gallbladder lesions for specimen collection [176]. Brush cytology is an invasive technique that may be useful as a screening procedure; however, this technique does not replace tissue biopsy. Therefore, more sensitive and non-invasive methods are needed in order to distinguish between patients with or without gallbladder lesions. Emerging evidence has shown that miRNA expression might be altered during various pathological conditions and may become non-invasive and specific molecular diagnostic or prognostic markers for human pathology [177]. This is mainly because they have great stability in various types of body fluids, including blood, urine, tears, colostrum, breast milk, bronchial lavage, amniotic, pleural, seminal, peritoneal and cerebrospinal fluids [178]. Furthermore, they are resistant to endogenous RNase digestion, and the laboratory detection methods are sensitive [179,180]. The first study to determine the presence of circulating miRNAs was conducted by Lawrie *et al.* [181] in 2008 on patients with diffuse large B-cell lymphoma, finding that high miR-21 expression was associated with relapse-free survival [181]. The role and expression of miRNAs in BTCs have been gradually explored; various miRNAs have been detected initially in tissues. In GBC, multiple miRNAs have been reported as downregulated compared to normal tissues, such as miR-1, miR-145 [21], miR-135a-5p, miR-26a [182,183], miR-34a [184], miR-335 [185], miR-130a [186] and miR-218-5p [187], and others miRNAs have been upregulated, such as miR-155 [188], miR-20a [189] and miR-182 [190]. In addition, some of these miRNAs (miR-26 [191], miR-34a [131] and miR-155 [192]) also exhibited differential expression in CCA. Meanwhile, miR-21, miR-141 and miR-200b are highly overexpressed in CCA cells, and inhibition of miR-21 and miR-200b is associated with sensitivity to gemcitabine [22,131]. Furthermore, miR-21 showed 95% sensitivity and 100% specificity in distinguishing between CCA and normal tissues [22]. Selaru *et al.* [22] determined in 2009 an expression profile of miRNAs in primary extrahepatic CCA; several miRNAs (miR-106b, miR-21, miR-107, miR-106a, miR-93, miR-27a, miR-19a, miR-103, miR 17-5p, miR-25) were found overexpressed, whereas expression of miR-560, miR-370, miR-198, miR-188, miR-662, miR-191, miR-512-3p, miR-520e, miR-513 and miR-494 was repressed [22]. Similarly, Chen *et al.* [133] identified a more significant expression profile in intrahepatic CCA, correlating mir-200C, miR-141, miR-223 and miR-204 with multifocal cholangiocarcinoma and vascular invasion. There is less scientific evidence in AC tissues, but a microRNA profile has been identified that would allow ampullary adenocarcinomas to be subclassified into pancreatobiliary or intestinal type [23]. Furthermore, miR-215 is the most significantly overexpressed in AC, whereas miR-134 and miR-214 were significantly lower in AC than in pancreatic carcinomas [193]. These unique expression profiles in tissue miRNAs could probably correlate with circulating levels and may be excellent candidates for biomarkers in the serum, plasma and bile of these patients. However, as observed in different studies, the expression of miRNAs in tissue in matched plasma was not always the same; for example, Plieskatt *et al.* [40], found that the expression of the eight miRNAs was detected exclusively in all iCCA plasma samples and not in control plasma and tissue samples. Conversely, 15 highly deregulated miRNAs in tumor tissue were absent in paired plasma samples, deregulated exclusively in tumor tissue. Similarly, Wang *et al.* [134] demonstrated that miR-150 was upregulated in iCCA plasma patients, observing an opposite result to matched tissue. As reported by other publications (not matched samples), for example, Shigehara *et al.* [114] demonstrated that in bile miR-145* was significantly upregulated in CCA and GBC compared to patients diagnosed with choledocholithiasis; however, our work published in 2014 found that in tissue, miR-145 was downregulated in GBC, compared to non-neoplastic tissues [21]. This may reflect different functions present in tissue and in circulating miRNAs. One of the mechanisms through which circulating miRNAs originate is active secretion via exosomes [107], where they are encapsulated in the extracellular vesicles in circulation blood and bile. It has been found that liver and biliary tree cells communicate through EVs and extracellular vesicle-transported miRNA species [194]. This suggests an active mechanism to selectively export or import miRNAs. It is an interesting route that could explain this phenomenon, since the miRNA profiles of exosomes may differ in tissue and plasma [195]; therefore, the expression level could vary from tissue and blood samples. This is consistent with that reported by Pigati *et al.* [196], who hypothesized the existence of an intrinsic mechanism of selection within cells for miRNA secretion, explaining the different cellular and extracellular miRNA profiles. Thereby, the secreted miRNAs do not necessarily reflect the amount of miRNAs in the origin cell [196].

In contrast, miR-192 levels were significantly higher in the serum and tissues of CCA patients; however, no significant difference was found in tissues [135]; miR-224 was significantly upregulated in serum, as well as in tissue from CCA patients [144]; and circulating mir-21 is upregulated in GBC [132] and CCA [30], as well as in the tissue of CCA patients [197]. Interestingly, it has been reported that miR-21 is highly overexpressed in GBC cell lines (NOZ) treated with aquaporin-5 (AQP-5) siRNA, and genetic defects involving aquaporin genes have been associated with GBC [198].

miR-21 has been characterized as playing an important role in BTCs [30,132], and it has been suggested that it could have a potential role as in early diagnostic biomarker. Previous reports have also shown that circulating miR-21 expression increases in inflammatory conditions [199]. However, in studies by Kishimoto *et al.* [30] and Cheng *et al.* [139], plasma miR-21 was capable of distinguishing BTC patients from BBD. Distinguishing BTCs and BBD is very difficult to achieve by detecting the standard tumor markers, such as CA19-9 [200]. The determination of the primary disease site in advanced stages is key for applying the appropriate treatment and obtaining the desire results in survival; however, this determination is currently too challenging to achieve without invasive diagnostic methods. On the other hand, miR-21 may lack specificity, because it is one of the most commonly deregulated circulation miRNAs in diseases such as liver cancer [201], esophageal cancer [202], prostate cancer [203], head and neck cancer [204], gastric cancer [205], lung cancer [206], lymphoma [181] and colorectal cancer [207]. Therefore, the use of single and/or multiple miRNA expressed as a profile/pattern and combined with other classic biomarkers measured in plasma/serum could provide more specific and relevant information to diagnose and predict BTCs. Thus, a single biomarker might not reflect the complexity of a disease, and a multimarker strategy may improve the sensitivity and specificity at diagnosis and prognosis. For example, Li *et al.* [24] defined a panel combining five miRNA markers for CCA diagnosis, and Shigehara *et al.* [114] demonstrated that the combination of two miRNAs (miR-9 and miR-145*) is a suitable diagnostic biomarker for BTCs. Meanwhile, Kishimoto *et al.* [30] showed that diagnostic power increases by using a combination of CA19-9 and miR-21 to differentiate BTC patients from healthy volunteers and BBD patients. Similarly, Wang *et al.* [134] showed that the diagnostic value is increased when miR-150 expression and CA19-9 are combined, improving patient screening. In contrast, Voigtlander *et al.* [136] showed that the diagnostic value is not increased by using a panel of miRNAs to differentiate patients with PSC from CCA.

In this manuscript, distinct expression patterns of circulating miRNAs have been observed, including some inconsistencies in the results. It is likely that the inconsistency in some data reported in the literature is due to ethnic variations [208]. Li *et al.* [24] delineated a different miRNA pattern to detect CCA in bile, including a multiethnic population (Caucasian, African American, Asian and Hispanic) in the analysis. Considering this variable and although most cases are Caucasian, this analysis is probably more representative of the general population. Considering that miRNAs may be circulating in different forms (freely circulating, bound to proteinsor enclosed in EVs) [108], multiple purification strategies should be considered to enable work with groups of circulating miRNAs separately, thus avoiding a biased group of miRNAs potentially having completely different physiological functions. Li *et al*. [24] and Que *et al.* [145] studied miRNA species solely isolated from EVs with no consideration of the soluble fraction of bile. In addition, RNAs extracted from free-floating cells in bile [24] and isolated from clinical samples of bile and biliary brushings have been shown to be highly degraded [209]. Meanwhile, due to the technical feasibility of obtaining biological material, many researchers cannot evaluate the expression of miRNAs in paired samples and obtain tissue from healthy patients (with no pre-neoplastic lesion), and as the bile collection is an invasive technique, healthy patients do not undergo biliary brushings by ERCP for use as a healthy control and to establish a baseline, which makes the analysis of this complex epigenetic mechanism limited and difficult. In the same context, most studies used samples from “healthy volunteers” as controls, and other studies used samples from benign bile-duct disease (BBD) as controls. Therefore, unfortunately, the expression profile of microRNAs in different preneoplastic stages (hyperplasia, metaplasia and dysplasia) is not comparable. Moreover, BBD controls are formed by large heterogeneous groups with an interesting biological diversity. For instance, Kishimoto *et al.* [30] included patients with gallstones, cholecystitis, adenomyomatosis, gallbladder polyp, cholangitis, choledocal cyst, malfusion of pancreaticobiliary duct, bile duct dysplasia and postoperative stenosis in their study. Similarly Li *et al.* [24] included patients with benign biliary obstruction, primary sclerosing cholangitis, sphincter of Oddi dysfunction, chronic pancreatitis and cholangitis as bile control. Meanwhile, in other reports [139], the histological grade of lesions was not informed. Other factors, such as the standardization of the collecting, transporting and storing the bile sample, as well as the molecular technique and its methodological variables (housekeeping gene), are factors that influence miRNA expression. When considering the minimum amount of sample (whole blood, bile, plasma or serum) required to perform the exRNA extraction, Yang *et al.* [210] established a reliable method to detect circulating miRNAs in 100 μL of blood sample in bile duct-ligated (BDL) mice; this is important in systemically-compromised patients, where sample collection can be complex prior to analysis in the laboratory. Other reports are: Kishimoto *et al.* [30] extracted total RNA from 400 μL of plasma; Plieskatt *et al.* [40] used 200 μL of plasma; Voigtländer *et al.* [136] used approximately 0.5–5 mL of bile sample; and Li *et al.* 400 μL of bile [24].

## 7. Concluding Remarks

In the context of cancer biology, as with other malignancies, BTCs exhibit an aberrant expression of miRNAs with a marked difference according to the stage and carcinogenic model. Table 2 summarizes all of those circulating miRNAs with the potential to become diagnosis biomarkers due to their significant deregulation in BTCs compared to healthy controls or benign lesions. Some of these miRNAs have the potential to become biomarkers for BTC diagnosis, such as miR-21, miR-187, miR-202 miR-483-5p, miR-505-3p, miR-150, miR-1281, miR-126, miR-26a, miR-200c, miR-9, miR-145*, miR-105, miR-106 and miR-224, as well as to predict prognosis, for instance, miR-187, miR-202, miR-143, mir-21, mir-192 and miR-106a. Specifically, miR-191, miR-486-3p, miR-1274b, miR-16 and miR-484 detected in bile specimens were clinically useful for noninvasive CCA diagnoses of PSC and benign biliary obstruction (BBO) [24], and miR-192 and miR-106a were found to be related to CCA aggressiveness and survival [135,139]. Moreover, miR-21 expression correlates with tumor progression [30], and a significant relationship was found between miR-187, miR-143 and miR-202 and lymphatic metastasis/TNM [132]. Collectively, these data reflect that circulating miRNAs could be used as potential biomarkers for clinical use, for the early detection and prognosis of BTCs [211]. In this context, the content of miRNAs [108] has characteristics according to the ideal biomarker, such as: accessibility, high sensitivity and specificity, early detection of disease and comparatively long half-life [212]. However, to date, few studies have specifically addressed the significance of circulating miRNAs in BTC patients, and further research is required.

## Figures and Tables

**Table 1:** Area under the curve (AUC), sensitivity and specificity values of miRNAs in plasma and bile samples, according to different histological conditions of biliary tract cancers.

miRNAs	AUC	Sensitivity	Specificity	Reference
BTCs *vs.* HVs				Kishimoto *et al.* [30]
miR-21	0.93	85.1%	100%	
BTCs *vs.* BBD				
miR-21	0.83	72.3%	91.3%	
iCCA *vs.* controls				Wang *et al.* [134]
miR-150	0.764	80.6%	58.1%	
iCCA *vs.* HVs				Silakit *et al.* [135]
miR-192	0.803	74%	72%	
PSC *vs.* CCA (serum)				Voigtlander *et al.* [136]
miR-1281	0.83	55%	90%	
miR-126	0.87	68%	93%	
miR-26a	0.78	52%	93%	
miR-30b	0.78	52%	88%	
miR-122	0.65	32%	90%	
PSC *vs.* CCA (bile)				
miR-412	0.81	50%	89%	
miR-640	0.81	50%	92%	
miR-1537	0.78	67%	90%	
miR-3189	0.80	67%	89%	
PSC *vs.* HVs				Bernuzzi *et al.* [137]
miR-200c	0.74	--	--	
CCA *vs.* HVs				
miR-483-5p	0.77	--	--	
miR-194	0.74	--	--	
miR-483-5p and miR-194	0.81	--	--	
CCA *vs.* PSC				
miR-222	0.71	--	--	
miR-483-5p	0.70	--	--	
miR-222 and miR-483-5p	0.77	--	--	
PBC *vs.* HVs				Kojima *et al.* [138]
Combination of eight	0.953	80.3%	97.6%	
miRNAs (miR-6075, miR-4294, miR-6880-5p, miR-6799-5p, miR-125a-3p, miR-4530, miR-6836-3p, and miR-4476)				
BTCs *vs.* choledocholithiasis				Shigehara *et al.* [114]
miR-9	0.975	88.9%	100%	
miR-145*	0.975	77.8%	100%	
miR-944	0.765	77.8%	100%	
CCA *vs.* HVs				Cheng *et al.* [139]
miR-106a	0.89	81.6%	85%	

CCA: Cholangiocarcinoma; HVs: Healthy volunteers; iCCA: Intrahepatic cholangiocarcinoma; PSC: Primary sclerosing cholangitis; BBD: Benign bile-duct disease; PBC: Pancreato-biliary cancers; AUC: Area under the curve; --: No data.

**Table 2:** Circulating miRNAs with potential to become markers in biliary tract cancers.

miRNAs: Up-/Down-Regulation	Samples Number	Type of Sample	Diagnosis/Prognosis Potential	Origin of Specimen	Relevance	Reference
Up: miR-21, miR-187, and miR-202 Down: let-7a, miR-143 and miR-335	GBC (40); HVs (40)	Plasma	All diagnosis/only miR-187, miR-202 and miR-143 prognosis	China	GBC *versus* HVs	Li *et al.* [132]
Up: miR-21	BTCs (94) including CCA, GBC and AC; HVs (50); BBD (23)	Plasma	Diagnosis and prognosis	Japan	BTCs *versus* HVs and BTCs *versus* BBD	Kishimoto *et al.* [30]
Up: miR-483-5p, miR505-3p, miR874, miR885-5p, miR-320b, miR-92b-3p, miR1275 and miR1307-3p	iCCA (13); HVs (5)	Plasma	Diagnosis	Thailand	iCCA *versus* HVs	Plieskatt *et al.* [40]
Up: miR-150	iCCA (15)	Plasma	Diagnosis	China	iCCA *versus* controls	Wang *et al.* [134]
Up: miR-192	iCCA (51); HVs (32)	Serum	Diagnosis and prognosis	Thailand	iCCA *versus* HVs	Silakit *et al.* [135]
Up (serum samples): miR-1281, miR-126, miR-26a, miR-30b and miR-122 Down (bile samples): miR-412, miR-640, miR-1537 Up (bile samples): miR-3189	PSC (40 serum,52bile); CCA (31 serum, 19 bile); PSC/CCA (12 bile); HVs (12 serum)	Serum/bile	Diagnosis	Germany	PSC *versus* CCA	Voigtlander *et al.* [136]
Down: miR-200c (PSC *vs.* HVs) Up: miR-483-5p and miR-194 (CCA *vs.* HVs) Up: miR-222 and miR-483-5p (CCA *vs.* PSC)	CCA (70); PSC (70); HVs (70)	Serum	Diagnosis	Italy	PSC *versus* HVs; CCA *versus* HVs; CCA *versus* PSC	Bernuzzi *et al.* [137]
Down: miR-125a-3p and miR-6893-5p	BTCs (98) including iCCA, eCCA, GBC, HBD and AC; HVs (150)	Serum	Diagnosis	Japan	BTCs *versus* HVs	Kojima *et al.* [138]
Up: miR-191, miR-486-3p, miR-1274b, miR-16 and miR-484	CCA (46); Control group (50) including BBO, PSC, SOD, CP and cholangitis	Bile in EVs	Diagnosis	USA (Including Caucasian;African American; Asian and Hispanic)	CCA *versus* PSC and BBO	Li *et al.* [24]
Up: miR-9, miR-145 *, miR-105, miR-147b, let-7f-2*, let-7i*, miR-302c*, miR-199a-3p, miR-222* and miR-942	BTCs (9) including CCA and GBC; Choledocholithiasis (9)	Bile	Diagnosis	Japan	BTCs *versus* choledocholithiasis	Shigehara *et al.* [114]
Down:miR-106a Up: miR-21 (no significant difference)	CCA (103); BBD (34); HVs (20)	Serum	Diagnosis and Prognosis	China	CCA *versus* BBD and HVs	Cheng *et al.* [139]
Down: miR-21 and miR-17-5p (no significant difference)	PC (22); AC (6); HVs (8)	Serum in EVs	--	China	AC *versus* PC	Que *et al.* [145]
Up: miR-210, miR-200c, miR-194 and miR-192 Down: miR-133a and miR-891a	Choledocholithiasis and HVs	Gallstones	--	China	Choledocholithiasis *versus* HVs	Yang *et al.* [152]
Up: miR-224	CCA (30); HVs (50)	Serum	Diagnosis	China	CCA *versus* HVs	Huang *et al.* [144]

CCA: Cholangiocarcinoma; GBC: gallbladder cancer; AC: ampulla Vater cancer; HVs healthy volunteers ; iCCA: intrahepatic cholangiocarcinoma; PSC: primary sclerosing cholangitis; eCCA: extrahepaticcholangiocarcinoma; HBD: hilar bile duct cancer; BBO: benign biliary obstruction; SOD: sphincter of Oddi dysfunction; CP: chronic pancreatitis; BBD: benign bile-duct disease; PA: pancreatic adenocarcinoma; EVs: extracellular vesicles; --: No data.

## Abbreviations

3′UTR	3′ Untranslated region
ABCA1	ATP-binding cassette transporter A1
AC	Ampulla Vater cancer
AGO	Argonaute
AQP-5	Aquaporin-5
AUC	Area under the curve
BBD	Benign biliary diseases
BBO	Benign biliary obstruction
BDL	Bile duct-ligated
BTCs	Biliary tract cancers
CA19-9	Carbohydrate 19-9
CCA	Cholangiocarcinoma
CEA	Carcinoembryonic antigen
CP	Chronic pancreatitis
CT	Computed tomography
CYP7A1	Cholesterol 7a-hydroxylase mRNA
dCCA	Distal cholangiocarcinoma
eCCA	Extrahepatic cholangiocarcinoma
ERCP	Endoscopic retrograde cholangiopancreatography
EUS	Endoscopic ultrasound
EVs	Extracellular vesicles
EXOmotif	miRNAs into exosomes attached to specific motifs
exRNA	Extracellular RNA
FXR	Farnesoid X receptor
GBC	Gallbladder cancer
HBD	Hilar bile duct cancer
HDL	High-density lipoprotein
hnRNP	Ribonucleoprotein
hnRNPA2B1	Heterogeneous nuclear ribonucleoprotein A2/B1
HVs	Healthy volunteers
iCCA	Intrahepatic cholangiocarcinoma
LncRNAs	Long non-coding RNA
LOH	Loss of heterozygosity
LXR	Liver X receptors
miRNAs	microRNAs
miRNA*	Temporary string miRNA
MRI	Magnetic resonance imaging
NOZ	Gallbladder cancer cell lines
PA	Pancreatic adenocarcinoma
PBC	Pancreato-biliary cancers
pCCA	Perihilarcholangiocarcinoma
pre-miRNA	Precursor miRNA
pri-miRNA	Primary miRNA
PSC	Primary sclerosing cholangitis
PTC	Percutaneous transhepatic cholangiography
RISC	RNA-induced silencing complex
SOD	Sphincter of Oddi dysfunction
TNM	Staging system for classification of malignant tumours
TUTases	Uridylyltransferases
XPO5	Exportin 5
